# Association Between Maternal Pre-Pregnancy Body Mass Index and Growth Delay in Korean Children Aged 18–36 Months: A Population-Based Study

**DOI:** 10.3390/jpm15060261

**Published:** 2025-06-19

**Authors:** Eun-Jung Oh, Tae-Eun Kim, Sang-Hyun Park, Hye Won Park, Hyuk Jung Kweon, Jaekyung Choi, Jinyoung Shin

**Affiliations:** 1 Department of Family Medicine, Konkuk University Medical Center, Chungju Hospital, Konkuk University School of Medicine, Chungju 27376, Republic of Korea; oej98@hanmail.net (E.-J.O.); fmkhj@kku.ac.kr (H.J.K.); 2 Department of Clinical Pharmacology, Konkuk University Medical Center, Seoul 05030, Republic of Korea; tekim@kuh.ac.kr (T.-E.K.); 20230583@kuh.ac.kr (S.-H.P.); 3 Department of Pediatrics, Konkuk University Medical Center, Konkuk University School of Medicine, Seoul 05030, Republic of Korea; 20110673@kuh.ac.kr; 4 Department of Family Medicine, Konkuk University Medical Center, Konkuk University School of Medicine, Seoul 05030, Republic of Korea; cjk@kuh.ac.kr

**Keywords:** body mass index, obesity, growth, development, administrative claims, health care

## Abstract

**Background:** Maternal pre-pregnancy body mass index (BMI) has been linked to childhood growth. However, its effects on growth delay at different early life stages are not well understood. This study aimed to evaluate the relationship between maternal pre-pregnancy BMI and growth delay in Korean children, using data from the National Health Screening Program for Infants and Children. **Methods**: Data from 258,367 children born between 2014 and 2021 who underwent health screenings at both 18–24 and 30–36 months of age were analyzed. Maternal BMI within three years before childbirth was classified into five categories: <18.5, 18.5–22.9 (reference), 23–24.9, 25–29.9, and ≥30 kg/m^2^. Growth delay was defined as measurements below the 10th percentile for height, weight, and head circumference. Adjusted relative risks (RRs) were estimated using regression models controlling for maternal age, comorbidities, and perinatal factors. **Results**: An increased risk of height growth delay was observed with higher maternal BMI, and this association persisted at both 18–24 and 30–36 months. In contrast, maternal underweight was not significantly associated with a height delay. Low maternal BMI was associated with underweight status in children. Head circumference growth delay was linked to both high and low maternal BMI; children of mothers outside the normal BMI range had an increased risk. **Conclusions**: Maternal pre-pregnancy obesity and underweight were associated with growth delays in height, weight, and head circumference in children up to 36 months of age. These findings underscore the importance of individualized weight management before pregnancy.

## 1. Introduction

Pre-pregnancy overweight and obesity are recognized as risk factors for adverse maternal and neonatal outcomes, including preterm birth, large for gestational age, and fetal overgrowth [1,2]. In South Korea, the prevalence of obesity among women has increased across nearly all age groups over the past decade, with the most significant increase observed among women in their 20s and 30s. Between 2013 and 2022, the obesity prevalence in women in their 20s increased 1.8-fold (from 10.3% to 18.7%) and 1.5-fold in women in their 30s (from 15.4% to 22.8%) [3]. Maternal weight across the childbearing period has been shown to influence the body composition and growth trajectories of offspring not only in early childhood (ages 2–5 years), but also during later stages including middle childhood (6–11 years), and adolescence (12–19 years) with a >2-fold increase in the risk of obesity [4]. Globally, childhood overweight and obesity have also been on the rise, contributing to an increased risk of noncommunicable diseases such as type 2 diabetes, cardiovascular disease, cancer, and mental illness later in life [5]. These conditions are thought to be influenced by early growth patterns characterized by restricted growth in utero, followed by rapid weight gain in early childhood [6]. According to the Developmental Origins of Health and Disease hypothesis, the first 1000 days of life constitute a critical window in which environmental exposures, including maternal nutritional status, can shape long-term health trajectories through epigenetic programming [5,6].

The National Health Screening Program for Infants and Children (NHSPIC) was initiated in South Korea in 2007 with the aim of supporting the normal growth of infants and children by monitoring and managing their physical growth and developmental milestones. The program is provided free of charge to all newborns nationwide, ensuring universal access, regardless of socioeconomic status. In the first year of implementation, the participation rate was 35.3%, and the detection rate of growth delays in height, weight, and head circumference ranged from 4.4% to 9.8% [7].

Existing research on the relationship between maternal body mass index (BMI) and childhood growth patterns has predominantly focused on Western populations, predominantly investigating the association between maternal BMI and childhood overweight (85–94th percentile) or obesity (≥95th percentile) [5]. In the Healthy Growth Study of 2666 Greek preadolescents aged 9–13 years, maternal pre-pregnancy BMI was associated with a 4.16-fold increased risk of childhood obesity (95% CI: 2.47–7.02), whereas gestational weight gain showed a smaller effect (OR 1.50, 95% CI: 1.08–2.08) [8]. These findings suggest that maternal pre-pregnancy BMI may exert a stronger influence on a child’s developmental trajectory. Few studies have explored whether maternal BMI prior to pregnancy is also associated with suboptimal growth indicators such as short stature, underweight, or reduced head circumference in early childhood. In an analysis of a Chinese birth cohort, offspring of underweight mothers (BMI < 18.5) exhibited lower BMI and smaller early growth increases during the first two years of life compared to those born to mothers with a reference BMI (18.5–23.9) [9]. Interestingly, maternal obesity (BMI ≥ 28) was not significantly associated with offspring growth, and the authors attributed this finding to the relatively low prevalence of maternal obesity in Southwest China, leaving the causal relationship unclear.

To date, there is a paucity of large-scale, population-based studies examining the association between maternal weight status prior to pregnancy and indicators of growth restriction in early childhood. Consequently, this study sought to explore the relationship between maternal pre-pregnancy BMI and the risk of growth delay in offspring using nationally representative health screening data for infants and children.

## 2. Materials and Methods

### 2.1. Study Data Sources and Participants

This study was organized as a retrospective cohort analysis based on data from the National Health Examination and the National Health Screening Program for Infants and Children (NHSPIC), provided by the National Health Insurance Service (NHIS). The NHIS conducts the National Health Examination every two years for all individuals aged 20 years and older, collecting self-reported medical histories and anthropometric measurements. The assessments were performed by survey and physical examinations at seven different intervals between 4 and 71 months of age (chronological age): the first visit occurred at 4–6 months of age, the second at 9–12 months, the third at 18–24 months, the fourth at 30–36 months, the fifth at 42–48 months, the sixth at 54–60 months, and the seventh at 61–71 months [10]. The NHIS database contains comprehensive information on diagnoses, procedures, and prescriptions for the entire South Korean population, identified using International Classification of Diseases, Tenth Revision (ICD-10) codes.

**Figure 1:**
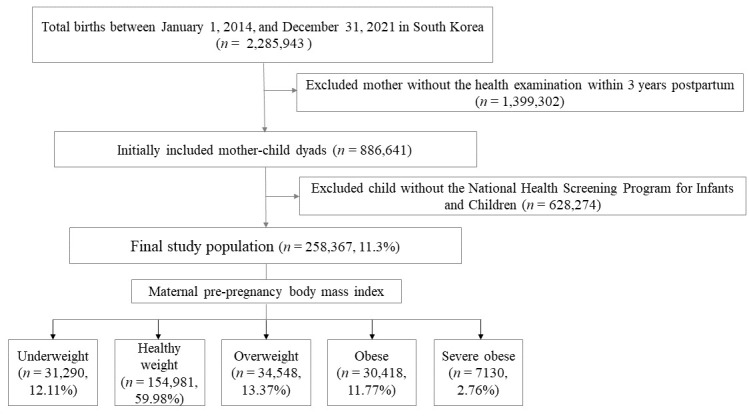
Study population.

Among 2,285,943 births from 1 January 2014 to 31 December 2021, 886,641 mothers who underwent the National Health Examination from the NHIS within three years post-childbirth and 779,091 children who participated in the NHSPIC were linked using the family’s unique health insurance card number and delivery date. For the final analysis, 258,367 mother-child pairs who attended both 18–24 and 30–36 months were included (Figure 1).

The study was conducted in accordance with the Declaration of Helsinki and approved by the Institutional Review Board (IRB) of this institution (KUMC: 2023-10-023). The requirement for informed consent was waived because this study did not include information on identifying individuals using previously collected data released to the general public.

### 2.2. Measurements

Maternal BMI was calculated as weight divided by the square of height (kg/m^2^) and categorized into five groups according to Asia-Pacific criteria: underweight (BMI < 18.5), healthy weight (BMI 18.5–22.9), overweight (BMI 23.0–24.9), obese class I (BMI 25.0–29.9), and obese classes II and III (BMI ≥ 30.0; severe obesity) [11]. For children aged 18–24 months, the recumbent length was measured by two trained examiners using an infantometer. The child was positioned supine with the head secured and the legs gently extended, ensuring that the heels made contact with the footplate. The measurements were recorded to the nearest 0.1 cm. For children aged 30–36 months, the standing height was measured without footwear. The child stood upright with a proper posture, and the headpiece was lowered to lightly compress the hair. Body weight was measured to the nearest 0.1 kg while the child wore light underwear with diapers removed. Head circumference was measured using a flexible, non-stretchable tape while the child looked straight ahead. The tape was placed around the most prominent parts of the forehead and occiput. Height and head circumference measurements were recorded to the nearest 0.1 cm.

Growth delay was defined as a height, weight, and head circumference below the 10th percentile. Although the World Health Organization Growth Standards employ standard deviation scores for children under 36 months and percentiles for those aged 3 years and older, the NHSPIC adapted the use of percentile values for children under 3 years as well to reduce confusion due to differences in criteria interpretation. [7]. In the NHSPIC, growth delay is typically defined as short stature and microcephaly below the 3rd percentile and underweight below the 5th percentile, according to age-specific standards. However, for the purpose of early identification of at-risk children, this study defined growth delay as values below the 10th percentile [12].

### 2.3. Other Variables

Maternal comorbidities were assessed as follows: hypertension (I10–I15 and O10, prescription of antihypertensive medication, or systolic/diastolic blood pressure ≥140/90 mmHg), pregnancy-induced hypertension (ICD-10 code: O12-15), diabetes mellitus (O24.0–O24.3, E100–E109, E110–E119, E120–E129, E130–E139, and E140–E149), gestational diabetes mellitus (GDM) (O24.4, O24.9), and depression (F329, F332, and F530). Delivery methods, including normal delivery or cesarean section, were identified using O800–O809, O820–O829, R435, R436, RA314–318, RA36–38, RA43, R314, R450, R451, R4520, and R500, as obtained from the claims data. Gestational age at birth, sex, birth weight, multiple births, preterm birth defined as gestational age at delivery earlier than 37 weeks, major anomalies (Q00–Q98.4), and small for gestational age (SGA) defined as birth weight below the 10th percentile (P050, P051, and P059), and neonatal intensive care unit (NICU) admissions post-birth (AJ101, AJ111, AJ121, AJ131, AJ144, AJ161, AJ201, AJ211, AJ221, AJ231, AJ244, AJ261, AJ301, AJ311, AJ321, AJ331, AJ351, AJ051~AJ054) were obtained.

### 2.4. Statistical Analysis

Continuous variables were presented as mean ± standard deviation, while categorical variables were expressed as numbers (proportions). Baseline characteristics were evaluated based on maternal BMI using analysis of variance (ANOVA) and chi-square tests. To account for initial differences in baseline characteristics across groups, we calculated the propensity score (PS) to equalize baseline covariates between the comparator and reference groups using a multivariable logistic regression model that incorporated all specified covariates. Subsequently, we determined the PS-weighted relative risks (RRs) of growth delays and 95% confidence intervals (CIs) using a generalized linear model [13]. Participants with a healthy BMI served as the reference group. These analyses were adjusted for factors such as maternal age, comorbidities, multiple births, major anomalies, birth weight, sex, SGA, preterm birth, delivery method, and admission to the intensive care unit post-birth. Crude incidence rates (IRs) per 1000 individuals with developmental delays were calculated. All statistical analyses were performed using SAS (version 9.4; SAS Institute, Inc., Cary, NC, USA).

## 3. Results

### 3.1. Study Population According to Maternal Pre-Pregnancy BMI

The study included 258,367 participants categorized according to maternal pre-pregnancy BMI as underweight (*n* = 31,290, 12.11%), healthy weight (*n* = 154,981, 59.98%), overweight (*n* = 34,548, 13.37%), obese (*n* = 30,418, 11.77%), and severely obese (*n* = 7130, 2.76%) (Table 1). Maternal age, chronic hypertension, pre-gestational and gestational diabetes, and pregnancy-induced hypertension increased significantly with higher BMI (*p* < 0.001). Concurrently, the rate of normal delivery significantly decreased (*p* < 0.001). Furthermore, as maternal BMI increased, there was a corresponding increase in birth weight, multiple births, and NICU admissions, along with a higher incidence of macrosomia, defined as birth weight ≥ 4000 g (*p* < 0.001) [14]. Notably, the prevalence of preterm births and major anomalies was the lowest among healthy individuals. Although the incidence of SGA was relatively low in the obese group, it increased significantly in the underweight group and cases of severe maternal obesity.

### 3.2. The Risk of Growth Delay

Table 2 presents the outcomes of growth delay at 18–24 and 30–36 months according to the maternal pre-pregnancy BMI categories. The risk of height growth delay increased with a higher maternal pre-pregnancy BMI. Although the difference in risk compared to the normal BMI group appeared to decrease slightly at 30–36 months, height growth delays remained consistently observed in the children of mothers classified as overweight, obese, or severely obese at 18–36 months of age. In contrast, maternal underweight was not significantly associated with height growth delay. Low maternal BMI was associated with an increased risk of underweight in children, whereas maternal obesity was associated with a decreased risk of underweight status in the offspring. Head circumference growth delay was associated with both high and low maternal BMI. Although the risk among children of obese mothers decreased slightly at 30–36 months, the risk of head circumference delay remained elevated in all groups, except those born to mothers with normal pre-pregnancy BMI.

## 4. Discussion

This study investigated the correlation between maternal pre-pregnancy BMI and growth delays by analyzing a large-scale dataset representing 11.3% of all births in Korea from 2014 to 2021. Underweight in the pre-pregnancy period was associated with an increased risk of underweight and reduced head circumference in the offspring. Conversely, maternal overweight and obesity were linked to an increased risk of growth delay in height and head circumference, but were associated with a reduced risk of growth delay in weight.

Individual variability in growth is influenced by multiple factors, including genetics, prenatal growth conditions, underlying morbidities, and nutritional status [15]. Although many SGA infants exhibit appropriate catch-up growth during the postnatal period, those who do not experience proper catch-up growth increase the risk of developmental delays and behavioral problems extending from preschool to school age, even among full-term infants [16,17]. In particular, compared to height or weight, head growth provides a more sensitive and valuable marker as a reliable indicator of brain growth for identifying potential neurological concerns and monitoring neurodevelopmental outcomes [15,18].

Previous studies have reported that maternal underweight is associated with adverse fetal outcomes, such as intrauterine growth restriction and low birth weight, which can lead to poor postnatal growth, including reduced head circumference and underweight status in infancy and early childhood [19]. Our findings align with these results, as maternal underweight was associated with an increased risk of underweight and smaller head circumference in children with increased adverse perinatal outcomes [20]. However, no significant association was observed between maternal underweight and delayed height growth in our study. This finding suggests that height growth is non-linear during early childhood. Individual differences in postnatal catch-up growth in height are more likely to be influenced by environmental factors than genetics, compared to weight and head circumference [21]. Therefore, a low maternal pre-pregnancy BMI did not significantly affect height growth delay.

High maternal BMI has traditionally been linked to large birth weights; however, emerging studies have also shown an increased risk of SGA births among offspring of obese mothers, suggesting a more complex, potentially U- or J-shaped relationship between maternal adiposity and fetal growth patterns [22]. In early childhood, our study showed that maternal obesity was associated with delayed growth in height and head circumference, with a possible dose-dependent pattern. This is consistent with prior research, including that of Kayla et al., who also reported significant associations between maternal BMI and early child growth, although our study did not adjust for maternal lifestyle factors or socioeconomic status [5].

These results emphasize the importance of precision management in early life care, in which prenatal risk stratification and individualized postnatal support can be informed by maternal characteristics. Maternal BMI is one of the strongest predictors of childhood growth and the developmental trajectory. Furthermore, maternal genetic factors contribute 22% to the variation in birth weight and 19% in birth length and head circumference from the parent-offspring cohort in the Medical Birth Registry of Norway (1967–2004), underscoring the importance of accounting for maternal genetic influence [23]. Pre-pregnancy BMI increased the risk of neurodevelopmental disorder for their children (overweight: OR = 1.17, 95% CI: 1.11, 1.24, and obese: OR = 1.51, 95% CI: 1.35, 1.69) in a meta-analysis for 32 studies [24].

Incorporating this factor into predictive models may enhance early identification and timely intervention efforts by accounting for the intrauterine environment, shaped by maternal metabolic and inflammatory status, and the early postnatal environment, where maternal behaviors related to nutrition, physical activity, and caregiving exert significant influence during critical developmental windows [6].

This study has several limitations. First, the analysis employed pre-pregnancy BMI as recorded in the health records; however, BMI is an imperfect indicator of nutritional status or body composition. The association between maternal pre-pregnancy BMI and fetal health outcomes may vary across continents when region-specific BMI classifications [25]. BMI does not account for fat distribution or micronutrient deficiency. Although gestational weight gain was not measured in this study, existing literature suggests that perinatal factors such as gestational weight gain, gestational age, and birth weight do not mediate these associations [26]. Korean women demonstrated an average gestational weight gain of 13.44 kg (SD 4.5). Interestingly, there was no significant variation in gestational weight gain across different pre-pregnancy BMI categories [27], emphasizing that pre-pregnancy BMI may exert a greater influence on offspring outcomes than weight gain during pregnancy. Second, this study was unable to comprehensively control for all potential confounding variables. Factors, such as socioeconomic status, nutrition, and education, which may influence child growth or maternal BMI, have not been thoroughly investigated. A systematic review demonstrated that breastfeeding exerts a protective effect against childhood obesity, and various infant feeding practices may also impact early development [5]. Finally, developmental assessments were conducted between 18 and 36 months of age, which is relatively early in a child’s life. Some children with early developmental delays may catch up over time with appropriate intervention, whereas others may encounter challenges later, even if they initially develop typically. Therefore, long-term follow-up is crucial to ascertain whether this observation is sustained.

## 5. Conclusions

Pre-pregnancy BMI in mothers is linked to delays in the growth of height, weight, and head circumference in children aged 18–36 months. The results of this study have important implications for individualized maternal health policies, highlighting the need for women of childbearing age to maintain a healthy BMI before pregnancy to reduce the risk of growth delay. Additional research is needed to investigate the impact of changes in maternal weight and conduct long-term studies that account for personalized risk factors.

## Figures and Tables

**Table 1:** Baseline characteristics of the study population.

Maternal BMI	Underweight	Healthy Weight	Overweight	Obese	Severe Obese	*p*-Value
N	31,290	154,981	34,548	30,418	7130	
%	12.11	59.98	13.37	11.77	2.76	
Maternal characteristics						
Age, mean, year	31.59 ± 3.69	32.23 ± 3.84	32.75 ± 4.06	32.96 ± 4.12	33.1 ± 4.26	<0.001
Age (≥35 years)	6219(19.88)	39,910(25.75)	10,953(31.7)	10,300(33.86)	2588(36.3)	<0.001
Chronic hypertension	445(1.42)	2523(1.63)	761(2.2)	1163(3.82)	682(9.57)	<0.001
PIH	2673(8.54)	15,462(9.98)	4235(12.26)	4458(14.66)	1669(23.41)	<0.001
Depression	581(1.86)	2638(1.7)	580(1.68)	606(1.99)	173(2.43)	<0.001
Pregestational DM	66(0.21)	553(0.36)	311(0.9)	622(2.04)	446(6.26)	<0.001
Gestational DM	4109(13.13)	22,093(14.26)	5921(17.14)	6436(21.16)	2059(28.88)	<0.001
Preterm birth	1365(4.36)	6554(4.23)	1464(4.24)	1382(4.55)	412(5.78)	<0.001
Normal delivery	20,162(64.44)	89,601(57.81)	17,480(50.6)	13,333(43.83)	2304(32.31)	<0.001
Children’s characteristics						
Gestational age, mean, week	35.6 ± 2.3	35.5 ± 2.3	35.3 ± 2.5	35.2 ± 2.5	34.8 ± 2.6	<0.001
Sex, male	15,957(51.0)	79,252(51.1)	17,664(51.1)	15,415(50.7)	3555(49.9)	0.180
Birth weight, mean, kg	3.09 ± 0.41	3.17 ± 0.43	3.23 ± 0.45	3.27 ± 0.47	3.31 ± 0.53	<0.001
Birth weight, kg						<0.001
<2.00	345(1.1)	1552(1)	371(1.07)	313(1.03)	110(1.54)	
2.00~2.99	10,633(33.98)	41,003(26.46)	7733(22.38)	6268(20.61)	1350(18.93)	
3.00~3.99	19,879(63.53)	108,432(69.96)	25,045(72.49)	22,060(72.52)	4983(69.89)	
≥4.00	433(1.38)	3994(2.58)	1399(4.05)	1777(5.84)	687(9.64)	
Multiple birth	998(3.19)	5715(3.69)	1239(3.59)	1086(3.57)	303(4.25)	<0.001
Preterm birth	1365(4.36)	6554(4.23)	1464(4.24)	1382(4.55)	412(5.78)	<0.001
Major anomaly	2613(8.35)	12,330(7.96)	2841(8.22)	2620(8.61)	698(9.79)	<0.001
SGA	351(1.12)	1306(0.84)	253(0.73)	183(0.60)	59(0.83)	<0.001
ICU admission	1800(5.75)	9065(5.85)	2202(6.37)	2191(7.20)	703(9.86)	<0.001

Data are presented as numbers (%) or mean ± standard deviation. BMI: body mass index, PIH: pregnancy-induced hypertension, DM: diabetes mellitus, SGA: small for gestational age, ICU: intensive care unit.

**Table 2:** The risks of growth delay in children of 18–24 months or 30–36 months, according to the maternal pre-pregnancy BMI.

		18–24 Months				30–36 Months			
**BMI**	**N**	**Event**	**IR**	**RR(95% CI)**	***p*** **-Value**	**Event**	**IR**	**RR(95% CI)**	***p*** **-Value**
Height < 10 percentile									
Underweight	31,290	1181	3.77	0.965(0.929–1.002)	0.065	1397	4.46	1.014(0.979–1.050)	0.438
Healthy weight	154,981	5242	3.38	1(ref.)		6074	3.92	1(ref.)	
Overweight	34,548	1214	3.51	1.133(1.092–1.175)	<0.001	1450	4.20	1.165(1.126–1.205)	<0.001
Obese I	30,418	1174	3.86	1.332(1.285–1.380)	<0.001	1328	4.37	1.274(1.233–1.317)	<0.001
Obese II&III	7130	335	4.70	1.610(1.556–1.667)	<0.001	332	4.66	1.310(1.267–1.355)	<0.001
Weight < 10 percentile									
Underweight	31,290	1748	5.59	1.223(1.183–1.265)	<0.001	1864	5.96	1.338(1.294–1.383)	<0.001
Healthy weight	154,981	5988	3.86	1(ref.)		5944	3.84	1(ref.)	
Overweight	34,548	1070	3.10	0.916(0.883–0.950)	<0.001	1093	3.16	0.923(0.890–0.957)	<0.001
Obese I	30,418	926	3.04	1.001(0.966–1.038)	0.948	912	3.00	0.989(0.954–1.026)	0.558
Obese II&III	7130	187	2.62	0.861(0.828–0.894)	<0.001	178	2.50	0.791(0.760–0.822)	<0.001
Head circumference < 10 percentile									
Underweight	31,290	1625	5.19	1.068(1.034–1.104)	<0.001	1855	5.93	1.111(1.077–1.146)	<0.001
Healthy weight	154,981	6528	4.21	1(ref.)		7298	4.71	1(ref.)	
Overweight	34,548	1367	3.96	1.031(0.997–1.067)	0.073	1536	4.45	1.036(1.004–1.069)	0.029
Obese I	30,418	1216	4.00	1.132(1.096–1.170)	<0.001	1296	4.26	1.072(1.038–1.106)	<0.001
Obese II&III	7130	339	4.75	1.375(1.332–1.419)	<0.001	332	4.66	1.191(1.154–1.228)	<0.001

BMI, body mass index; RR, relative risk; CI, confidence interval. Incidence rates were calculated per 1000 people. Adjusting for maternal age, comorbidities, multiple births, major anomalies, birth weight, sex, SGA, preterm birth, delivery method, and admission to the intensive care unit post-birth.

## Data Availability

The data supporting this article are accessible from the National Health Insurance Service Open Data Portal.

## Abbreviations

BMI	Body mass index
NHSPIC	National Health Screening Program for Infants and Children
NHIS	National Health Insurance Service
ICD-10	International Classification of Diseases, Tenth Revision
IRB	Institutional Review Board
SGA	Small for gestational age
NICU	Neonatal intensive care unit
CIs	Confidence intervals
IRs	Incidence rates
